# Comparison of Three Modelling Approaches for Predicting Deoxynivalenol Contamination in Winter Wheat

**DOI:** 10.3390/toxins10070267

**Published:** 2018-07-02

**Authors:** Cheng Liu, Valentina Manstretta, Vittorio Rossi, H. J. van der Fels-Klerx

**Affiliations:** 1 RIKILT Wageningen University & Research, Akkermaalsbos 2, 6708 WB Wageningen, The Netherlands; cheng.liu@wur.nl; 2 Horta srl, via Egidio Gorra 55, 29122 Piacenza, Italy; v.manstretta@horta-srl.com; 3 Department of Sustainable Crop Production (DI.PRO.VE.S.), Università Cattolica del Sacro Cuore, Via Emilia Parmense 84, 29122 Piacenza, Italy; vittorio.rossi@unicatt.it

**Keywords:** DON, cereal grains, food safety, forecast, mycotoxin, validation

## Abstract

Forecasting models for mycotoxins in cereal grains during cultivation are useful for pre-harvest and post-harvest mycotoxin management. Some of such models for deoxynivalenol (DON) in wheat, using two different modelling techniques, have been published. This study aimed to compare and cross-validate three different modelling approaches for predicting DON in winter wheat using data from the Netherlands as a case study. To this end, a published empirical model was updated with a new mixed effect logistic regression method. A mechanistic model for wheat in Italy was adapted to the Dutch situation. A new Bayesian network model was developed to predict DON in wheat. In developing the three models, the same dataset was used, including agronomic and weather data, as well as DON concentrations of individual samples in the Netherlands over the years 2001–2013 (625 records). Similar data from 2015 and 2016 (86 records) were used for external independent validation. The results showed that all three modelling approaches provided good accuracy in predicting DON in wheat in the Netherlands. The empirical model showed the highest accuracy (88%). However, this model is highly location and data-dependent, and can only be run if all of the input data are available. The mechanistic model provided 80% accuracy. This model is easier to implement in new areas given similar mycotoxin-producing fungal populations. The Bayesian network model provided 86% accuracy. Compared with the other two models, this model is easier to implement when input data are incomplete. In future research, the three modelling approaches could be integrated to even better support decision-making in mycotoxin management.

## 1. Introduction

Mycotoxin contamination in cereals has been recognised as a global threat for human and animal health, especially under the pressure of climate change [1,2]. Deoxynivalenol (DON) produced by *Fusarium* spp. (mainly *Fusarium graminearum* and *Fusarium culmorum*) is one of the most studied and frequently occurring mycotoxin in winter wheat globally. Contamination of wheat with *Fusarium* spp. and DON is influenced by the wheat variety, agronomic practices (e.g., tillage), and local weather, especially around wheat flowering. Monitoring and predictive modelling have become some of the solutions for farmers, collectors, and food safety authorities to manage mycotoxin contamination during cereal cultivation and beyond. Some predictive models for mycotoxins have been developed and implemented in agricultural sectors in Europe as decision support systems for farmers [2,3,4,5,6,7,8]. These models have been developed by either an empirical or mechanistic modelling approach. In the past five years, models have been updated with recent monitoring data, and modelling techniques have been improved. For example, machine learning algorithms have recently been introduced in food safety areas [9,10]. Machine learning explores the study of pattern recognition and computational learning theory in artificial intelligence. Such algorithms can learn from sample inputs and make data-driven predictions or decisions [11]. Bayesian network (BN) modelling is one of many machine learning algorithms, and can possibly also be used to predict mycotoxin contamination in cereals. 

Logistic regression is a classic method to estimate the parameters of a logistic model, in which the log-odds of the probability of a binary event (e.g., presence or absence of mycotoxins) is a linear combination of independent variables [12]. Logistic regression is used in various areas, such as in medicines, social sciences, and life sciences. Mixed effects models contain both fixed effects (variables in the regression model) and random effects. With a hierarchical structure in the data (e.g., with repeated sampling in the same year/at the same farm), such a mixed model with random effects can improve the prediction accuracy.

A BN model is a probabilistic model that is based on Bayesian statistics and decision theory in combination with graph theory [13,14]. It is a directed graphical model that represents conditional probabilities among a set of variables, based on the application of the Bayes’ theorem. Compared to linear regression models, BN models can more easily analyze dependencies between variables, manage non-linear interaction, and combine different sorts of information, such as measurement data, expert knowledge, and end-user feedback [15]. 

Mechanistic models simulate the fundamental mechanisms of plant and fungus development stages and their interactions. Such models require well-developed knowledge of each biological process, as well as extensive experimental research under a wide range of conditions to collect the required knowledge and input data. 

This study aimed to compare and cross-validate three different modelling approaches, i.e., the empirical, BN, and mechanistic approach, for predicting DON contamination in winter wheat using data from the Netherlands as a case study. These models aim to support decision-making by farmers regarding limiting mycotoxin contamination by optimizing their agricultural management practices, such as fungicide sprays and harvest time. Grain collectors can also use the model predictions to buy wheat from low-risk areas in advance, or to optimize sampling plans for mycotoxin analysis. Food safety authorities can use the model predictions to locate high-risk areas for a more targeted and cost-efficient mycotoxin monitoring. Cross-validation of the three modelling techniques was done to further understand the pros and cons of each technique, and contribute to future research and progress in the prediction of mycotoxin contamination in food and feed. 

## 2. Results

### 2.1. Mixed Effect Logistic Regression Model

#### 2.1.1. Model C (for Farmers W0–W4)

Adding the random effect “year” improved the performance of model C for the DON medium/high contamination class (Akaike information criterion (AIC) differences are more than two), but not for the low/medium class. All of the variables were significant (*p* < 0.05). AIC values for low/medium class and medium/high class were 498.36 and 238.6, respectively. Results of model C indicated that the growing area (GArea) explained most of the variance in DON contamination, followed by cultivar resistance level (ResisL). The variance and standard deviation of the random effect year were 2.418 and 1.555, respectively.

**Figure 1:**
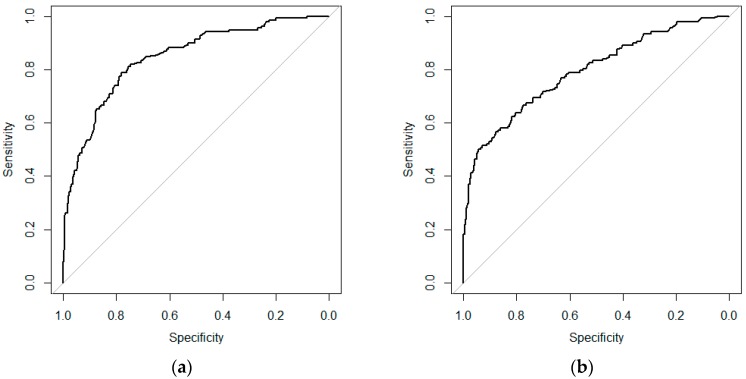
Mean receiver operating characteristic (ROC) curves for 10-fold cross-validation of Model C class low/medium (**a**), and Model C class medium/high (**b**).

Out of the 625 records for model development, 487 were classified as low, 81 were classified as medium, and 57 were classified as high for DON contamination. Model C correctly classified 511 out of these 625 observations (81.8%) (Table 1). Model C tended to underestimate (14.7%) rather than overestimate (3.5%) the DON contamination. Cross-validation analysis of Model C showed that the mean area under the curve (AUC) for the low/medium class was 84.3% (range 76.8% to 94.4%) (Figure 1a), and for the medium/high class was 80.1% (range 62.5% to 91.5%) (Figure 1b, indicating that Model C has a good prediction value.

#### 2.1.2. Model D (for Collectors W0–W6)

Model D results for the DON contamination of low/medium class had the same results as the Model C low/medium class. This result showed that weather conditions between flowering and harvest did not provide a statistically significant contribution to predict low levels of DON contamination. All of the variables were shown to be significant (*p* < 0.05). AIC for the medium/high class was 231.5. Similar to Model C, the results of Model D also indicated that the GArea explained most of the variance in DON contamination, followed by the ResisL. Variance and standard deviation of random effect year were 1.81 and 1.35, respectively.

**Figure 2:**
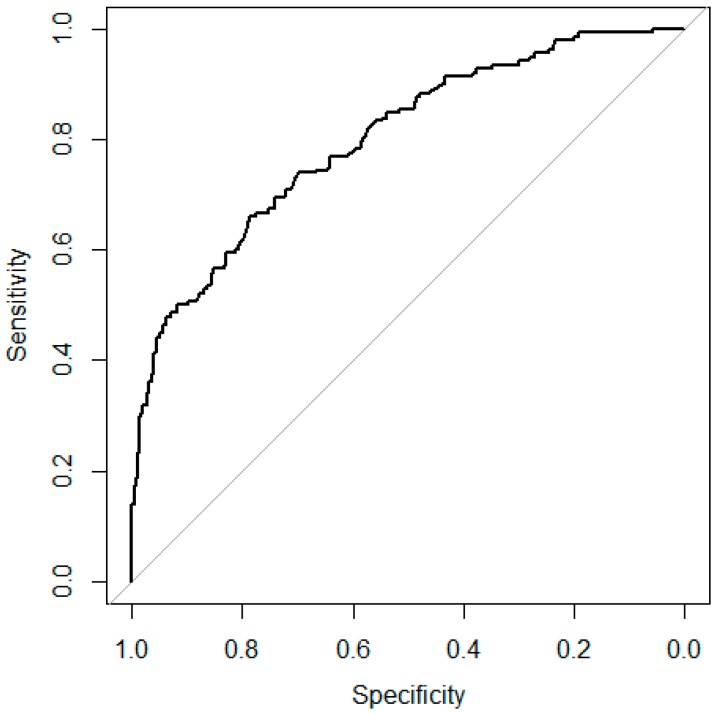
Mean receiver operating characteristic (ROC) curves for 10-fold cross validation on the Model D medium/high class.

Model D correctly classified 509 out of 625 observations (81.4%) (Table 2). Similar to Model C, Model D also tended to underestimate the DON contamination (15.2%), rather than overestimate it (3.4%). A cross-validation analysis of Model D showed that the mean area under the curve (AUC) for the DON low/medium class was the same as for the Model C low/medium class (Figure 1a), because the functions were the same. The cross-validation result for the DON medium/high class was 80.0% (range 72.3% to 89.1%) (Figure 2).

### 2.2. Bayesian Network Model

**Figure 3:**
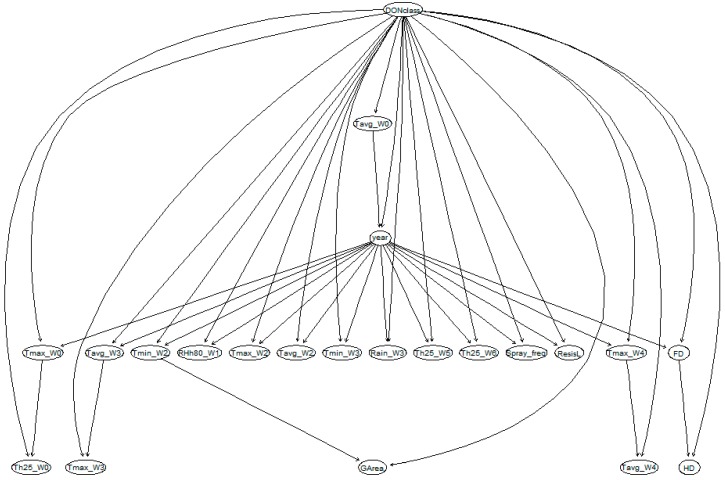
The Bayesian network model structure resulting from learning the model with field data collected during 2001–2013 in the Netherlands. Circles represent nodes of the Bayesian network model, and arrows indicate the relationship/conditional dependencies among the nodes. Tavg_W0–W4: average temperature in the time windows W0 to W4; Tmax_W0–W4; maximum temperature in the time windows W0 to W4; Tmin_W2–W3: minimum temperature in the time window W2 to W3 ; RHh80_W1: number of hours that relative humidity is higher than 80% in the time window W1; Th25_W0–W5: number of hours that average temperature is higher than 25°C in the time windows W0 to W5; Spray_freq: frequency of fungicides application around wheat flowering for controlling Fusarium head blight; FD: flowering date; HD: harvesting date.

In the BN model, the variables/nodes are shown as ellipses, and the relationship between the variables are indicated by directed arcs (Figure 3). In this BN model, DONclass is the parent of all of the other variables. The flowering date (FD) variable is called the child of DONclass if the link goes from DONclass to FD (Figure 3). The relationships between parent and child variables were quantified through a set of conditional probability tables (CPTs). Each variable was assigned a CPT given the information on its parents. An example of the calculated conditional probability of DONclass given the information of GArea is shown in Table 3. The BN model can provide predictions as soon as at least one variable is given as input. The marginal probability tables for each node are expressed in percentages of each level of the variables. The BN model in this study predicted the following distribution of probabilities for DONclass: low (78.1%), medium (12.8%), and high (9.1%). The BN model correctly classified 82.8% (Table 4) of the records. In case of misclassification, 7.7% of the cases were overestimated, and 9.4% of the cases were underestimated (Table 4). 

### 2.3. Mechanistic Model

In the separation of DON contamination into three groups, F1 accounted for 95.3% of the variation. So, it was decided to consider only the first discriminant function in order to have a simpler representation of the results. Canonical coefficients (CC), standardised canonical coefficients (SCC), and correlation coefficients (COC) collectively indicated that the GArea had a major effect in distinguishing the groups in F1, followed by the ResisL (Table 5).

Non-linear regression analyses for the probability of belonging to the low or high classes provided a good fit (Table 6). 

Discriminant function analysis (DFA) correctly classified 496 of the 625 samples (i.e., 79.4% of correct classification) (Table 7). In case of misclassifications, the real DON contamination was underestimated in 115 cases (18.5% of the total), while the remaining 14 (2.2%) cases were overestimated. 

### 2.4. Validation of Three Models

In the set of 87 records used for model validation, 82 were in class ‘low’, three were in the medium class, and two were in the high class for DON contamination (see Section 4.1 for class definitions). One sample had no information on the frequency of fungicides application around wheat flowering for controlling Fusarium head blight (Spray_freq), and was excluded from the data for validation of the regression models.

Mixed effect logistic regression (both Model C and Model D) correctly classified 76 of the 86 samples (88%) (Table 8). In this external validation, Model C and Model D both underestimated and overestimated 6% of the total samples. Model C did not predict the two samples in class ‘high’. 

The BN model correctly predicted 85% of the samples using all of the variables for prediction. Having less information would result in a lower accuracy of the prediction. As for Model C, the BN model did not predict the two samples in DON contamination class ‘high’.

DFA correctly classified 70 of the 87 samples (i.e., 80.5% of correct classification) (Table 8). In case of misclassifications, the real DON contamination was more severe than the predicted one in four cases (4.6% of the total), while DON contamination was overestimated for the remaining 13 samples (14.9%). Unlike Model C and the BN model, DFA was able to correctly classify one of the two samples in the high class. 

In summary, correct classifications from the regression model, BN model, and mechanistic model were 92.7%, 90.2%, and 84.1%, respectively, for low DON contaminations. None of the models predicted the three samples with a medium or high contamination, except for the mechanistic model, which predicted one of the two samples with a high DON contamination. 

## 3. Discussion and Conclusions 

This study compared the performances of three modelling approaches for predicting DON contamination in winter wheat by using Dutch data as a case study. Two of these models (the regression-based and the BN models) are new, while the third model (the mechanistic model) was adapted (included in a DFA) for being used with Dutch data. These models have been calibrated with the same dataset collected between 2001–2013 in the Netherlands, and cross-validated with another dataset collected in 2015 and 2016 in the Netherlands. From this exercise, the pro and cons of three modelling approaches are summarized, and results are discussed (Table 9).

Logistic regression is a classic and well-studied modelling approach that is applied in many research fields, including the food safety area. In this study, an evolution of a previously published empirical model [3] is presented. The updated model uses mixed effect logistic regression with recent field data, and predicts DON contamination classes instead of concentrations. Out of the three considered modelling approaches, mixed effect logistic regression models had the highest accuracy for low DON contamination, but not for medium and high contaminations. This method shows the statistical relationship between independent variables and the response variable collected in a specific agricultural context, which is the Dutch wheat-growing areas, in this case. Therefore, this method is highly data-dependent (Table 9), which implies that the regression parameter estimates cannot be used for other agricultural conditions than represented with the data used in model development, before proper validation. In addition, the model cannot be run in cases where data on one of the input variables (e.g., the resistance level of the variety) is missing. This implies that with the current models, predictions can only start from one week before FD (with using 10 days of weather forecast data). 

In this study, a new Bayesian network model to predict DON contamination at harvest in winter wheat has been developed. The BN model predicted all of the cases in the low DON contamination class, and had an accuracy of 94.3%. Similar to the regression models, the BN model was not able to correctly predict any of the DON contamination cases in the medium or high class from the validation dataset. The structure of the BN enables the model developer to easily add new information and/or expert knowledge in the model. BN models can use a combination of statistical relationships and expert knowledge (Table 9). The BN model is flexible, and can predict DON contamination even when only one variable is available. For instance, the BN model can calculate a base risk using GArea and ResisL at the beginning of the growing season. With these two variables, the BN model correctly predicted 81.6% of the cases (70 cases in the low class and one case in the high class) (data not shown). This largely extends the prediction possibilities to the start of the growing season. In this study, the BN model included all of the variables used in the regression models, in order to compare the two modelling approaches based on the same variables. In future studies, the best set of variables will be selected for the BN only.

The mechanistic model of Rossi et al. [16,17] was included in the DFA in order to classify DON contamination of winter wheat in the Netherlands. Similar to the other models, the mechanistic model and DFA were not able to correctly predict any DON contamination cases in the medium class, but the model predicted correctly one of the two cases in the high DON class. 

In summary, all three modelling approaches have good prediction accuracy in forecasting low DON contamination in winter wheat in the Netherlands, and are suitable for providing decision support to farmers and collectors. In the present work, the three modelling approaches were developed and tested using data mostly composed by cases in the ‘low’ class of DON contamination. Further validation is needed to confirm the accuracy of the model predictions in cases in which DON contamination is higher and close to the European Union (EU) legal limit. 

Implementing a mycotoxin forecasting model that has been developed for certain conditions, such as for one wheat-growing area or country, to other conditions as represented in the data used for model development, should always be done with utmost care and proper model testing and validation. From the experiences gained in our study, it was shown that the three modelling approaches each have their own advantages and disadvantages (Table 9).

Regression-based models and BN models are empirical. Being developed and parameterised based on a specific dataset, they represent the mathematical relationships among that data or similar conditions only. Therefore, empirical models should only be used for those conditions, such as the area/country, as represented by the observational data used for model development. Thus, these models are unsuitable to predict situations that did not occur in the model development dataset (Table 9), such as extreme weather events due to climate change or new agricultural practices (e.g., increase of conservation tillage or diffusion of new crop rotation types, which both affect the Fusarium head blight, or FHB, inoculum availability). BN models can include expert’s knowledge, and are very flexible regarding add new information to the forecasting system. In addition, BN models can produce output with incomplete information on the model’s input variables, even though this may lead to a reduction in the accuracy of their outputs. 

The mechanistic model, by definition, simulates the biological processes, which are in this case fungal infection, growth, and mycotoxin production. Such process-based models are not highly location-dependent, and are thus much easier to implement in other countries (Table 9). However, the mechanistic model has rather high requirements for data availability, and runs with specific types of data, such as heading date and leaf wetness duration, which are not always available. Furthermore, spatial differences on fungal species still need to be considered when implementing a mechanistic model in different wheat-growing areas. For instance, the frequency of *Fusarium* spp. and mycotoxigenic strains within species differs between North/Central Europe and South Europe [18]. Due to the low presence of *F. culmorum* in winter wheat grown in the Netherlands [19], only *F. graminearum* was considered in the current mechanistic model for the Netherlands, whereas both *F. graminearum* and *F. culmorum* were considered in the Italian model. 

Since all of the different modelling approaches have both pro and cons, a modelling ensemble in which different model types are combined could be interesting solution. Constructing such a modelling ensemble is part of the EC-funded MyToolBox project [20]. It will integrate the models in a unique framework and strengthen their advantages to provide more timely and accurate advice, in order to limit mycotoxin contamination and improve the safety of cereals and derived feed and food commodities. 

## 4. Materials and Methods 

### 4.1. Data 

**Figure 4:**
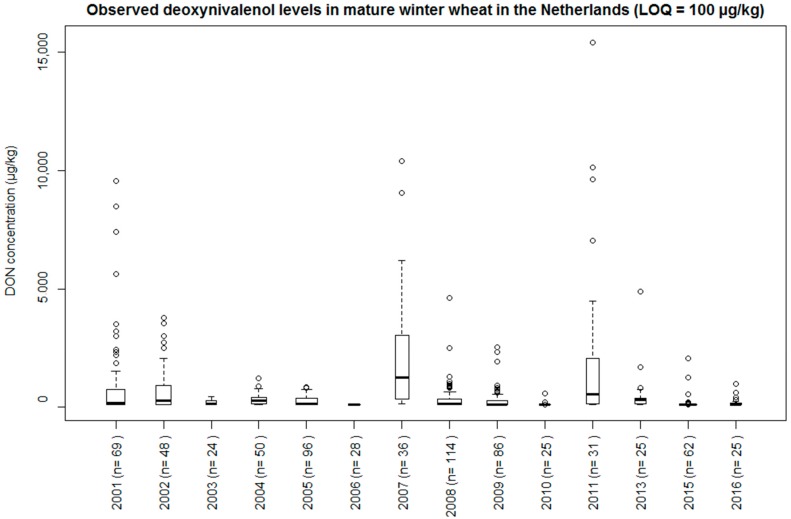
Observed deoxynivalenol (DON) concentrations in mature winter wheat in the Netherlands, 2001–2011, 2013, and 2015–2016. The midline (bold) of the box represents the median of the data, with the upper and lower limits of the box being the third and first quartile (75^th^ and 25^th^ percentile), respectively. The whiskers extend 1.5 times the interquartile range, from the top/bottom of the box to the furthest value within that distance. Data beyond that distance are represented individually as outliers (black circles). The box width is proportional to the square roots of the number of observations in that year. In all of the samples from 2006, the DON concentration was below the (highest) limit of quantification (100 µg/kg).

In the years 2001–2013 (except for 2012), mature winter wheat samples were collected from 632 fields (records) in the Netherlands (Figure 4). Agronomic information of these fields was also collected and included postal code, wheat variety, ResisL, Spray_freq, ploughing (yes/no), tillage method, pre-crop, observed FD, and observed harvesting date (HD). ResisL was divided into four groups with decreasing proneness to DON contamination: susceptible (5 and 5.5), medium susceptible (6, 6.5), medium resistant (7, 7.5), and resistant (8, 8.5). All of the agronomic information was collected by means of a farmer questionnaire. Unfortunately, not all of the agronomic information was available for all of the wheat fields. 

Weather inputs included hourly temperature, precipitation, and relative humidity from 25 automatic weather stations in the Netherlands. These data are freely available from the Royal Netherlands Meteorological Institute (KNMI) website (http://www.knmi.nl/nederland-nu/klimatologie/daggegevens). Then, each field was assigned to the nearest weather station based on postal codes. The average distance between a field and assigned weather station was 12 km, ranging from a minimum distance of <2 km to a maximum distance of 37 km. 

Samples of wheat kernels were collected at harvest from the combiner machine. Each sample contained 1 kg of mature winter wheat kernels. The samples were transported to the laboratory of RIKILT Wageningen University & Research. At RIKILT, DON was extracted and quantified using a liquid-chromatography with double in-line mass spectrometry (LC-MS/MS) [21]. The limit of quantification (LOQ) varied over the years from 5 µg/kg to 100 µg/kg. The highest LOQ was therefore used in further data analysis, and samples with DON concentrations below this LOQ were assigned the value of 100 µg/kg.

Observed FD and observed HD of the wheat field was not always recorded by the farmer. Therefore, the FD and HD of all of the winter wheat fields were calculated based on the sum of the daily average temperatures above 0 °C, using the method described in Van der Fels-Klerx et al. [3]. 

The variable GArea was used in the models to test the influence of the farm’s geographical location on the DON concentrations. GArea was calculated using geostatistical spatial analysis Kriging in ArcMap version 10.4.1 (Environmental Systems Research Institute, Redlands, CA, USA) [22]. Standardized DON concentrations ((DON value − average)/standard deviation) were used in the calculation [5]. Using kriging interpolation, seven standardized DON classes were calculated from low to high. GArea was then defined as seven cultivation areas in the Netherlands with a proneness or potential probability of DON contamination in wheat, which were further grouped into three classes (standardized DON values ≤0.08, 0.08–2, >2) as in Rossi et al. (2007) for model comparison. With this calculation, the GArea class at any unknown location was obtained as a weighted average of the standardized DON contamination in the neighborhood of the location.

The wheat-growing season was divided into seven time windows that were each one week in length, by using FD as day 0. Day 0 was defined as 1 pm on the previous day to 12 am on the current day. The weekly time windows started from 24 days prior to FD to four days after FD and one week around HD, and were defined as follows:

- W0: FD − 24 1 pm to FD − 17 12 am,
- W1: FD − 17 1 pm to FD − 10 12 am,
- W2: FD − 10 1 pm to FD − 3 12 am,
- W3: FD − 3 1 pm to FD + 4 12 am,
- W4: FD + 4 1 pm to FD + 11 12 am,
- W5: FD + 11 1 pm to HD − 3 12 am,
- W6: HD − 3 1 pm to HD + 4 12 am

Note that FD and HD were positioned in the middle of the time windows W3 and W6 to limit the influence of estimating FD and HD using growing degree days. All of the time windows consisted of seven days except for W5. The length of W5 (in days) varied between the wheat fields, because the time between FD and HD differed per location due to the differences in local weather. For each time window, the following weather variables were calculated:

- Tavg: Average hourly temperature in the time window (°C)
- Tmin: Minimum hourly temperature in the time window (°C)
- Tmax: Maximum hourly temperature in the time window (°C)
- P: Sum of the precipitation in the time window (mm)
- RHh80: Number of hours that relative humidity was higher than 80% (hour)
- Th25: Number of hours that temperature was higher than 25 °C (hour)

The regression model and the Bayesian network (BN) model were developed using R (version 3.3.2). The mechanistic model was developed using SPSS (version 24; IBM Corp., Armonk, NY, USA). All of the models predicted the probability of DON concentrations at harvest in three classes (DONclass): low (≤500 µg/kg), medium (500 µg/kg to 1250 µg/kg), and high (>1250 µg/kg). 

### 4.2. Mixed Effect Logistic Regression Model

The stepwise selection was performed combining significant (*p*-value < 0.25) weather and agronomic variables to predict DON concentrations in winter wheat. Year was added as a random effect to the model to test whether it improved model performance, as assessed by the Akaike information criterion (AIC). The farmers’ model (Model C) and the collectors’ model (Model D) were developed using weather variables till around the flowering period (W1–W4) and the entire cultivation period (W1–W6), respectively. 

Mixed effect linear regression models were developed in three steps: univariate analysis, stepwise logistic regression, and adding random effects. All of the statistical tests were assessed for significance at the 95% confidence level (*p* < 0.05), except for the univariate analysis (*p* < 0.25). Univariate analysis was performed following the method detailed in Van der Fels-Klerx [3]. The resulting agronomic and weather variables were considered in the stepwise logistic regression analyses. Then, year was added as a random effect to the model, in order to explain yearly variation in DON concentrations without using year as a prediction variable. The AIC was used to compare and select the best-fitting model. FD and HD were centred and scaled (dividing the centred data by their standard deviations) in the mixed effect model to limit the large differences in the scales of parameters, especially with calculating standard deviations of fixed effects. The pre-crop and tillage variables were not included in the model, because more than two-thirds of the records had no information about pre-crop or tillage. 

The final models calculated the probabilities of DON concentrations above 500 µg/kg as P1 and above 1250 µg/kg as P2. The probabilities of three DON classes were then calculated as: Low Class = 1 − P1, Mid Class = P2 − P1, and High Class = P2. The predicted class of each sample was finally defined as the class with the highest probability. 

To assess the model performance, a 10-fold internal cross-validation was conducted. Data for model development (2001 to 2013) were randomly divided into 10 subsets of equal size, and nine subsets were used for training the model, while the 10^th^ subset was used to test the model’s performance. This process was repeated 10 times, every time with a different test subset. So, all of the records were used for both training and testing, and each record was used for validation once. The mean area under the curve (AUC) was calculated. 

### 4.3. Bayesian Network Model

BN model development consists of four steps: (1) define variables of interests (also called notes), (2) structure learning, (3) parameter learning to calculate conditional probability tables (CPTs) for each parameter, and (4) BN validation. In order to compare different modelling approaches, a BN model was developed using the same variables as in the mixed effect linear regression models. The machine learning technique ‘Tree-Augmented Naive (TAN) Bayes’ learning algorithms was used to learn the BN model structure and compute the CPTs [23]. The structure and parameter learning steps were performed using the R package ‘bnlearn’ [24]. Continuous weather variables were discretised into discrete variables with eight clusters (same number of levels for variable year) by equal interval width, in order to ease and optimise the computation in the ‘bnlearn’ package. The validation step is detailed in Section 4.5.

In this setting, the relationships among all of the variables were constructed and indicated as directed edges. Note that these directed edges do not present the causal relationships, but rather only the statistical relationships among variables. 

### 4.4. Mechanistic Model

A mechanistic model for Fusarium head blight (FHB) and mycotoxin contamination, taking into account the effect of weather, was developed by Rossi et al. [16,17] using the system analysis approach [25,26]. Equations of the model were developed using data obtained from basic research on the biology and epidemiology of the main *Fusarium* species involved in FHB in Italy (i.e., *F. graminearum*, *F. culmorum*, *F. avenaceum*, and *M. nivale*). These studies were carried out under both controlled-environment and field conditions. As *F. graminearum* is the main species present in the Netherlands [19], the mechanistic model was adapted to consider this fungal species only.

**Figure 5:**
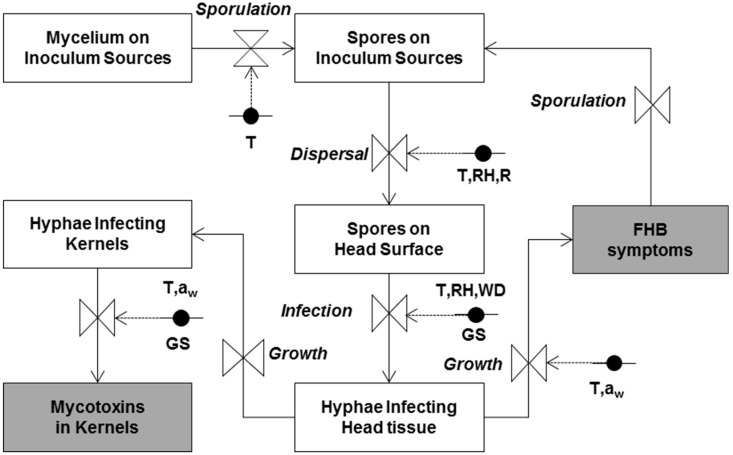
Simplified relational diagram of the Fusarium head blight (FHB) model predicting the probability of DON contamination in wheat. T = temperature; WD = leaf wetness duration; RH = relative humidity; GS = wheat growth stage; a_w_ = water activity; R = rainfall. Triangles: equations; Black circles: data inputs.

The simplified relational diagram of the model is shown in Figure 5. State variables (in rectangular boxes) represent the status of the pathogen at a given moment. The flow (arrows) from one state variable to another is regulated by rate variables (drawn as valves), and defined as the rate of change of the state variables in time as a function of driving variables, which are constants or parameters influencing these rate variables. Rates are regulated by mathematical equations describing the relationships between the rate and the (external) influencing weather and plant variables. Circles indicate variables influencing each rate, and are linked to valves by dotted arrows. The modelling steps are detailed in the original papers by Rossi et al. [16,17,26]. 

In a previous work [5,19], data for cropping practices collected in Italy in multi-annual surveys have been subjected to a cluster analysis based on the standardized DON concentration in the wheat sampled collected from the monitored fields. The categorical variables for wheat variety resistance and growing area have been combined with the model output (FHB-tox) by discriminant function analysis (DFA). Briefly, the procedure automatically selected a function (F1) that is able to separate the three classes of DON contamination as much as possible [27]. 

In this study, a new DFA was carried out on the (model development) data of the 625 fields collected in years between 2001–2013. DFA was used to determine the probability that each case belonged to each of the three DON contamination groups, determine group membership based on these probabilities, and obtain information on the effect of the discriminant variables on group membership. To determine group membership, the prior probability of group membership was set as proportional to the number of observations in each group (i.e., 0.78 for low, 0.13 for medium, and 0.09 for high DON contamination). For the effect of discriminant variables on group membership, the canonical coefficients (CCs) and standardised canonical coefficients (SCCs) were calculated. The magnitude of CCs and SCCs is an indicator of the weight of each variable in each of the discriminant functions. 

### 4.5. Validation of Three Models 

The three models were validated using field data collected in 2015 and 2016 in the Netherlands from 87 fields. These data were collected similarly as the data for model development from 2001 to 2013, as described in Section 4.1. These validation data have not been used previously for any model development or testing. Samples were classified for their DON contamination as previously described. For each of the three model types, the predicted probabilities of each sample in the DON classes of low, medium, or high were compared with the observed DON classes of low, medium, or high. 

For validation of the logistic regression models (Model C and Model D), the parameter coefficients of Model C and Model D (Section 2.1) were used to solve the logistic functions. The same method as described in Section 4.2 was used to calculate the probabilities of each of the three contamination classes. 

The BN model used the structure and conditional probabilities learned from the model development dataset for the prediction. Predictions of DONclass were tested given all of the information on agronomic factors. The parent note DONclass gave the probabilities of the three DON classes. 

DFA enables prediction of new cases based on the previously established structure. DFA was evaluated for the prediction of the DON contamination class (i.e., low, medium, or high) by using the FHB-tox index calculated at wheat harvest, and the values of the other discriminant variables for the 87 validation records. These data were used to solve the canonical discriminant function by using the non-standardised canonical coefficients calculated in Section 4.4. A non-linear regression was calculated between the value of the discriminant function for each sample (F1) and the corresponding probability (P(x)) to belong to the low or high class of DON contamination according to the following equation:

(1) P(x)=1/(1+exp(a−b×F1))

where a and b are equation parameters.

The probability for a field to belong to the medium class was calculated as the difference between one and the sum of the probabilities of the low and high classes.

Results of the fitting of Equation (1) were used to calculate the probabilities to assign new samples to the classes low, medium or high. 

## Figures and Tables

**Table 1:** Model C performance comparing predicted deoxynivalenol (DON) contamination classes versus observed DON classes (N = 625). 81.8% are correctly classified, 3.5% are overestimated, and 14.7% are underestimated by Model C.

Confusion Matrix	DONclass	Predicted		
		Low	Medium	High
Observed	Low	471	10	6
	Medium	70	5	6
	High	17	5	35

**Table 2:** Model D performance comparing predicted DON contamination classes versus observed DON classes (N = 625). 81.4% are correctly classified, 3.4% are overestimated, and 15.2% are underestimated by Model D.

Confusion Matrix	DONclass	Predicted		
		Low	Medium	High
Observed	Low	469	11	7
	Medium	70	8	3
	High	18	7	32

**Table 3:** Conditional probability of DONclass given the information of GArea.

		DONclass		
		Low	Mid	High
GArea	Green	0.835	0.117	0.048
	Yellow	0.677	0.175	0.148
	Red	0.363	0.050	0.587

**Table 4:** Bayesian network model performance comparing predicted DON contamination classes versus observed DON c classes (N = 625). 82.8% are correctly classified, 7.7% are overestimated, and 9.4% are underestimated.

Confusion Matrix	DONclass	Predicted		
		Low	Medium	High
Observed	Low	444	29	14
	Medium	45	31	5
	High	8	6	43

**Table 5:** Coefficients for discriminant variables in each canonical function (F1) used to classify the DON contamination (N = 625 samples) based on the mechanistic model output using Fusarium head blight (FHB)-tox, ResisL, and GArea as discriminant variables.

Discriminant Variables	Canonical Coefficient ^1^	Standardised Canonical Coefficient ^2^	Correlation Coefficient ^3^
GArea	1.837	0.905	0.837 *
ResisL	0.724	0.555	0.442
FHB-tox	0.075	0.073	−0.043
Constant	−4.429	-	-

^1^ Coefficients of the discriminant function. The discriminant function takes the form: F = a + b1 × GArea + b2 × ResisL + b3 × FHB-tox, where a is the constant, and bn are the canonical coefficients. ^2^ The standardized canonical coefficient is an indicator of the weight of each variable in the discriminant function. ^3^ The correlation coefficient indicates the discriminant power of each variable in each function. * indicates the largest absolute correlation between each variable and any discriminant function. Variables with correlation coefficient ≥0.3 are interpreted as important.

**Table 6:** Parameters and statistics of the equations describing the relationship between F1 and the probability of belonging to the DON contamination low or high classes.

	a ^1^	Standard Error	b	Standard Error	R^2^
Low	−1.455	0.009	−0.853	0.007	0.973
high	3.063	0.012	1.382	0.007	0.994

^1^ The regression equation was Equation (1) (see Section 4.4). a: constant; b: canonical coefficient.

**Table 7:** Mechanistic model performance comparing predicted DON contamination classes versus observed DON classes (N = 625). 76.5% are correctly classified, 2.2% are overestimated, and 18.4% are underestimated by the mechanistic model.

Confusion Matrix	DONclass	Predicted		
		Low	Medium	High
Observed	Low	478	0	9
	Medium	76	0	5
	High	39	0	18

**Table 8:** Comparison of predicted versus observed DON contamination class using the 87 samples collected in the years 2015 and 2016. One sample had no information on spray frequency, and was excluded from validation of the regression models.

		Predicted											
		Regression Model (for Farmers)			Regression Model (for Collectors)			BN Model			Mechanistic Model		
		Low	Mid	High	Low	Mid	High	Low	Mid	High	Low	Mid	High
Observed	Low	76	5	0	76	3	2	74	0	8	69	0	13
	Mid	3	0	0	3	0	0	3	0	0	3	0	0
	High	2	0	0	2	0	0	2	0	0	1	0	1

**Table 9:** Pros and cons of the regression model, Bayesian network model, and mechanistic model. DFA: discriminant function analysis.

	Regression Model	Bayesian Network Model	Mechanistic Model
Prediction accuracy low DON	93.8%	90.2%	84.1%
Prediction accuracy medium DON	0%	0%	0%
Prediction accuracy high DON	0%	0%	50%
Possibility to apply in other conditions (e.g., countries)?	High data dependency. Only in those countries/regions with similar agricultural and weather conditions. Validation needed before its use in new agricultural contexts	High data dependency. Only in those countries/regions with very similar agricultural and weather conditions. Validation needed before its use in new agricultural contexts	Low data dependency. The model can be implemented in other countries/regions given that the fungal species are similar. The combination of model output with influencing agronomic practices in a new country/region needs calibration through a specific DFA.
Prediction time	One week before flowering, using 10 days’ weather forecast data	From beginning of the growing season	From heading date
Capability to predict unknown situations	No	No	Yes
Requirement for specific data	Low	Low. Possible to combine expert knowledge with statistical relationships.	High, e.g., heading date, and leaf wetness duration.
